# Cross-cultural adaptation and validation of the original “diabetes basic knowledge test” (DBKT) into Brazilian-Portuguese version

**DOI:** 10.1186/1758-5996-7-S1-A184

**Published:** 2015-11-11

**Authors:** Layla Line Coutinho, Marcella Lobato Dias Consoli

**Affiliations:** 1 Instituto de Ensino e Pesquisa Santa Casa BH, Belo Horizonte, Brazil

## Background

To provide a reliable, validated, and culturally adapted instrument that may be used in evaluating the knowledge about diabetes in Brazilian health professionals.

## Materials and methods

The cross-cultural adaptation process of the original The Diabetes: Basic Knowledge Test (DBKT) into Brazilian-Portuguese was conducted using an appropriated guideline. First were made two translations, synthesis and back translation. The expert committee contributed on several steps. For validation, the adapted version was applied in a prospective longitudinal study to 105 health professionals with experience in education in diabetes mellitus less than six months and/or professional course carrier or expertise in education in diabetes. Internal consistency, reliability, and measure validity were assessed. Sample was predominantly made up of women (83.8%), aged between 22 and 67 yrs. (34.7±9.65 SD), with greater participation of nutritionists (42.9%), followed by doctors (20%) and nurses (18.1%). The adapted version of DBKT instrument has 41 items measured with a score of one (1) to the correct answer and zero (0) to incorrect. There is only one correct answer for each item.

## Results

About psychometric properties, the principal component analysis was conducted on 41 items of the instrument with orthogonal rotation (VARIMAX). The measure of Kaiser-Meyer-Olkin (KMO=0.604) verified the sample adequacy for analysis. Sphericity test of Bartlett (chi-square (104)=1386.12, p<0.001) indicated that the correlations between items are sufficient to perform the factor analysis. Cronbach's alpha value of the cross-culturally adapted Brazilian-Portuguese version of the DBKT was 0.81. The extraction criterion of the factors adopted by this study was the screeplot. This factor analysis, extracted from two factors, being Factor 1 (30 items) and Factor 2 (11 items). The mean of correct answers was 29.9±5.8 SD (maximum score: 41). About the Domain 1 was observed the mean was 20.9±5.1 SD (maximum score: 30). For Domain 2 the mean was 9.0±1.5 SD (maximum score: 11). Was not observed mean difference of the total scores for gender (p=0.58) and professional category (p=0.16).

## Conclusion

A well-established guideline resulted in a culturally adapted Brazilian-Portuguese version of the DBKT, tested and validated on a sample of Brazilian population, and proved to be a valid and reliable instrument for assessing the knowledge about diabetes in Brazilian health professionals.

